# Structural Characterization of an Archaeal Lipid Bilayer as a Function of Hydration and Temperature

**DOI:** 10.3390/ijms21051816

**Published:** 2020-03-06

**Authors:** Marta Salvador-Castell, Bruno Demé, Philippe Oger, Judith Peters

**Affiliations:** 1 Université de Lyon, INSA de Lyon, CNRS, UMR 5240, 69211 Villeurbanne, France; marta.salvador-castell@insa-lyon.fr; 2 Institut Laue Langevin, 38000 Grenoble, France; deme@ill.fr; 3 Université Grenoble Alpes, LiPhy, CNRS, 38000 Grenoble, France

**Keywords:** archaea, diphytanyl phospholipids, ether lipids, phase transition, neutron diffraction

## Abstract

Archaea, the most extremophilic domain of life, contain ether and branched lipids which provide extraordinary bilayer properties. We determined the structural characteristics of diether archaeal-like phospholipids as functions of hydration and temperature by neutron diffraction. Hydration and temperature are both crucial parameters for the self-assembly and physicochemical properties of lipid bilayers. In this study, we detected non-lamellar phases of archaeal-like lipids at low hydration levels, and lamellar phases at levels of 90% relative humidity or more exclusively. Moreover, at 90% relative humidity, a phase transition between two lamellar phases was discernible. At full hydration, lamellar phases were present up to 70 °C and no phase transition was observed within the temperature range studied (from 25 °C to 70 °C). In addition, we determined the neutron scattering length density and the bilayer’s structural parameters from different hydration and temperature conditions. At the highest levels of hydration, the system exhibited rearrangements on its corresponding hydrophobic region. Furthermore, the water uptake of the lipids examined was remarkably high. We discuss the effect of ether linkages and branched lipids on the exceptional characteristics of archaeal phospholipids.

## 1. Introduction

The archaea domain consists of unicellular microorganisms that have adapted to withstand extreme conditions, such as high temperatures, high hydrostatic pressures, and outermost pH values. All these environments represent a stress for the non-adapted organisms. For example, high temperatures would lead to an unstable, unreasonably permeable cell membrane and the loss of the physicochemical properties of the functional lipid phase. To sustain these extreme conditions, phospholipids that assemble the archaeal membranes are unique and differ from usual bacterial lipids. Archaeal lipids consist of an sn-glycerol-1-phosphate backbone, ether linkages, and phytanyl hydrocarbon chains, while most bacterial lipids include an sn-glycerol-3-phosphate backbone, ester linkages, and straight acyl chains [1,2]. Both phytanyl chains and ether linkages impact the physicochemical properties of lipids.

Phytanyls form methyl-branched chains that confer extraordinarily high stability to the archaeal cell membrane. The most studied diphytanyl lipid, 1,2-diphytanoyl-sn-glycero-3-phosphocholine (DPhPC), has proven to have a low permeability to ions and water and is highly resistant to oxidation [3,4,5]. For instance, its water permeability is about three times lower than its straight-chain homologue, 1,2-dipalmitoyl-sn-glycero-3-phosphocholine (DPPC) [5,6]. Moreover, DPhPC does not present a gel-to-liquid phase transition in a broad range of temperatures (−120 °C to 120 °C), probably because the methyl branches are equally spaced along the chains [7]. The equidistant localization of the methyl chains causes trans-gauche conformers to nearly be energetically equivalent, and hence gel-to-liquid transitions should be low-enthalpy processes. Moreover, the phytanyl chains favour the lateral interdigitation of hydrocarbon chains, which diminish the motion of the chains and provide unique structural stability [8,9].

Ether lipids have been investigated much less than DPhPC so far and only scarce information is available. For example, it is known that ether bonds are more stable than ester bonds, conferring higher resistance to hydrolysis [10]. Since ether oxygens are less polar than ester bonds, the solvation and hydrogen bonding of DPhPC and its ether analogue DoPhPC may be different, impacting on the mechanics and electrostatics of the interfacial layer [11]. Lastly, although phosphatidylcholine is the universal membrane component, in native membranes it is always accompanied by phospholipids with other headgroups such as phosphatidylserine, phosphatidylinositol, and phosphatidylethanolamine (PE). A change in the headgroup modifies electrostatic energies, water incorporation, and the gel-to-liquid transition temperature [12]. For example, since the choline head group possesses methyl groups, it does not allow close oxygen and nitrogen contacts, contrary to the ammonium group that forms strong electrostatic and hydrogen bonds between oxygen and nitrogen [13]. Consequently, PE lipids are stiffer than phosphocholine (PC) lipids, as demonstrated in diphytanoyl lipids [11]. Furthermore, the distinct headgroups modulate the bending modulus of the lipid bilayer inducing particular curvatures [14,15]. For example, while DPhPC is the only diphytanoyl lipid that produces stable lipid multilayers in oriented samples, DPhPE’s negative curvature is so important that the lipid alone self-assembles under non-lamellar phases, such as cubic and hexagonal phases [16]. Nevertheless, lipid mixtures containing DPhPC and up to 50% of DPhPE form unilamellar vesicles [13]. 

**Figure 1:**
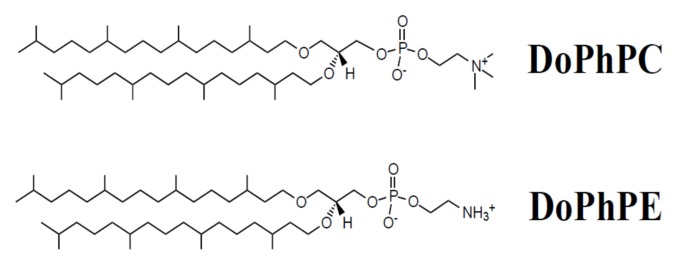
Sketch of the structures of the lipids used, 1,2-di-O-phytanyl-sn-glycero-3-phosphocholine (DoPhPC) and 1,2-di-O-phytanyl-sn-glycero-3-phosphoethanolamine (DoPhPE).

Due to the important effects of headgroups in the physical properties of lipid bilayers, in this study, we examined the effect of humidity and temperature on a mixture of archaeal-like lipid bilayers composed of 1,2-di-O-phytanyl-sn-glycero-3-phosphocholine (DoPhPC) and 1,2-di-O-phytanyl-sn-glycero-3-phosphoethanolamine (DoPhPE) (9:1) (see Figure 1). If lipids with a phosphocholine polar headgroup remained to be identified in archaea, those with a phosphoethanolamine headgroup were frequent in methanogens and archaeoglobales. This choice of archaeal lipid analogues was motivated by the fact that these synthetic lipids are commercially available as pure compounds, thus their composition is known exactly, they are not prone to non-reproducible mixtures, and they have suitable electrical charges. Temperature and hydration are usual experimental parameters to determine the physicochemical properties of the membrane and they are fundamental to determine the lipid bilayer characteristics. We used neutron diffraction to determine the membrane properties as it gives access to many structural parameters and permits us to relate them to hydration and temperature.

## 2. Results and Discussion

### 2.1. Hydration

**Figure 2:**
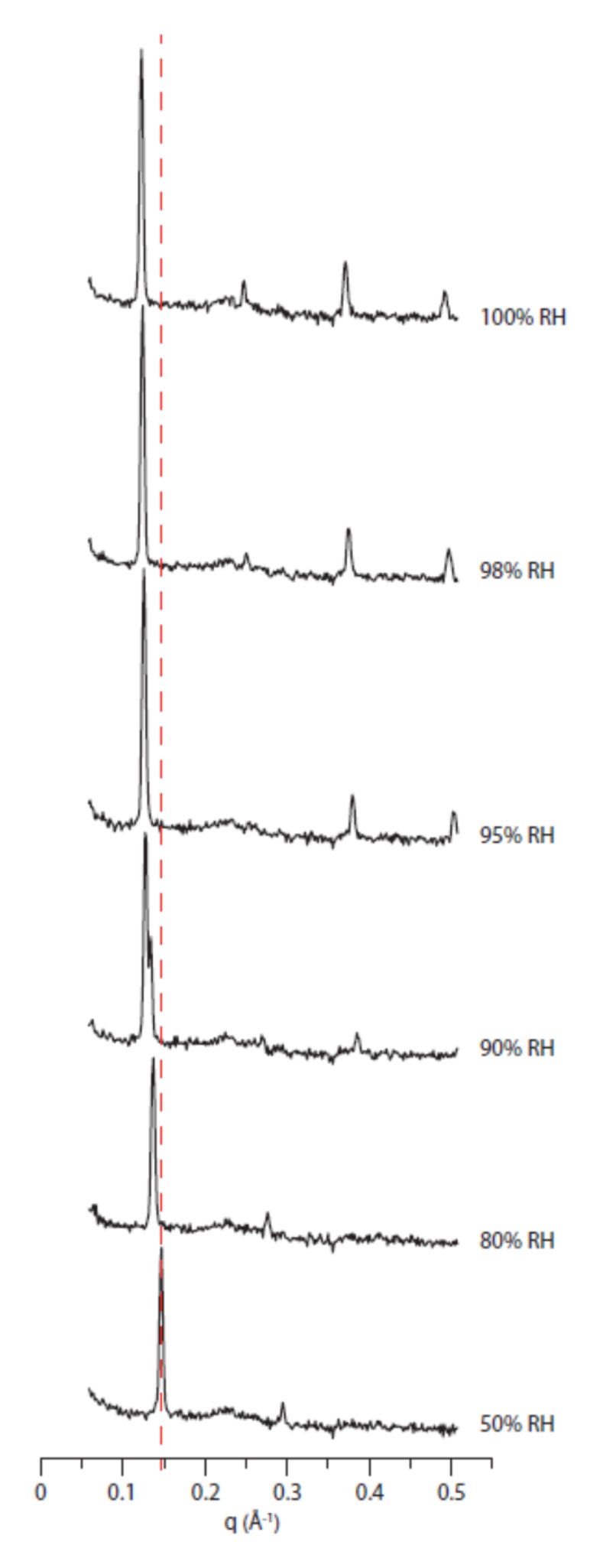
1D neutron diffractogram as a function of relative humidity (RH). DoPhPC:DoPhPE (9:1) diffractograms after background subtraction obtained by neutron diffraction at 25 °C as a function of relative humidity. The samples were hydrated with 8% D_2_O. The Y-axis intensity is (a.u.) at decimal log scale. The dotted line is a guide for the eye to show the Bragg peak shifts.

From the diffraction data, the q-values of Bragg peaks indicate the lattice parameter *D*, i.e., the distance between lipid bilayers including the water leaflet. As shown in Figure 2, higher levels of relative humidity (RH) shift Bragg peaks to lower q-values indicating an increase of *D*.

The 2D diffractograms of the mixture DoPhPC:DoPhPE (9:1) at 50% RH and 80% RH revealed the presence of a non-lamellar phase (Figure S1). The diffraction pattern of DoPhPC:DoPhPE (9:1) at 90% RH indicates an inhomogeneity of the sample due to the stable coexistence of two lamellar phases, which implies a phase transition (Figure 2). Previous studies have indicated similar phase transitions of DPhPC depending on hydration. For example, at 80% RH, Wu et al. detected a lamellar-to-lamellar phase transition [17] which was recently attributed to a lipid headgroup rearrangement [18], and Hsieh et al. found a marked polymorphism including non-lamellar phases, such as the hexagonal phase, under humidity levels lower than 80% RH [19]. 

The lipids hydrated above 90% RH arrange as a system self-organized under lamellar phases and up to four Bragg orders were easily detected. Nevertheless, these four orders, compared to the bacterial lipids which can commonly reach 10 orders [20], are indicative of a system with little order, probably due to their intrinsically disordered hydrocarbon chains. Although the phytanoyl chains were positioned perpendicularly to the bilayer plane similarly to the acyl chains of bacterial lipids, their distribution was broader, showing a higher disorder in the hydrophobic region [8,21]. A molecular dynamics simulation study from Husslein et al. (1998) even found that some of the chains may be parallel to the bilayer plane.

**Figure 3:**
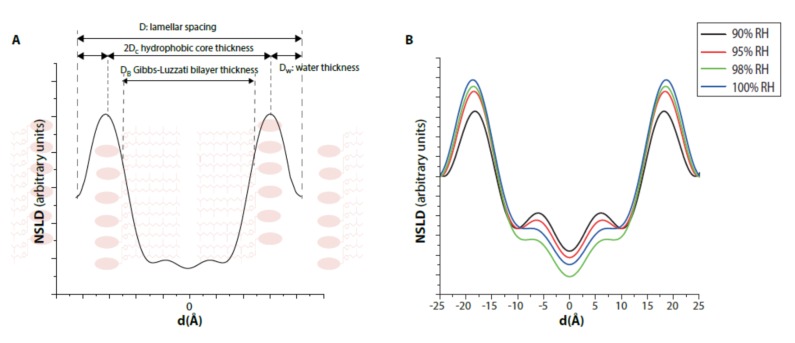
Neutron scattering length density (NSLD) as a function of relative humidity (RH). (**A**) Scheme presenting the thicknesses that can be extracted from the NSLD. (**B**) Lipids hydrated with 8% D_2_O. NSLD of DoPhPC:DoPhPE (9:1) at 25°C as a function of humidity: 90% RH (black), 95% RH (red), 98% RH (green), 100% RH (blue).

To get an insight into the lamellar phases observed at high humidity levels, we measured the neutron density profiles for such conditions (Figure 3). Neutron scattering length density (NSLD) profiles present the conventional shape for a lipid bilayer: a main peak from the headgroup region of the phospholipids, a central region that corresponds to the hydrophobic chains of the bilayer, and a minimum density from the terminal methyl. Interestingly, it seems that hydration between 90% RH and 98% RH changes the hydrocarbon conformation of the region from 10 Å to the bilayer midplane, although this region is highly impermeable to water molecules [22]. The global variation from 90% to 100% RH is related to the interactions existing within the lamellar phase and is difficult to disentangle. A higher order might have appeared with hydration, leading to more pronounced Bragg peaks in the diffractogram, but the different contributions to the interactions might also influence the thermodynamics differently. However, this is beyond the present study.

From the NSLD it was possible to extract the characteristic parameters of a lipid bilayer besides the lattice parameter (*D*) [20] (Table 1 and Figure 3A). The peak-to-peak distance from the NSLD indicates the Gibbs–Luzzati bilayer thickness (*D_B_*), 2*D_c_* corresponds to the thickness of the bilayer hydrophobic region, *D_w_* to the thickness of the water layer between lipid bilayer, and *n_w_* to the number of water molecules. The DoPhPC:DoPhPE mixture at 100% RH presented the following values: *D* = 50.4 ± 0.1 Å; *D_B_* = 37.4 ± 0.2 Å; 2*D_c_* = 31.4 ± 0.6 Å and *D_w_* = 13.0 ± 0.3 Å. The values obtained are in the order of the values obtained for DPhPC, for which *D_B_* was determined as 38 Å [17] and 35.4 Å [4] and 2*D_c_* as 27.2 Å [4] and 27.8 Å [23]. Once the lamellar phases were exclusively present, i.e., above 90% RH, the values were nearly unchanged, and the fluctuations of the lattice parameters were mainly due to a slightly thicker water layer at higher humidity levels. Interestingly, the uptake of the number of water molecules (16.2 ± 0.2) is much higher than in usual bacterial straight lipids (6.5 for DPPC [24]), and also slightly higher than in ester branched lipids (14.5 for DPhPC [25]), which was probably due to the presence of DoPhPE in the system. The values obtained for *D_w_* and *n_w_* changed only slightly with the RH level; however, both consistently pointed towards a continuous increase of the water layer.

### 2.2. Temperature

**Figure 4:**
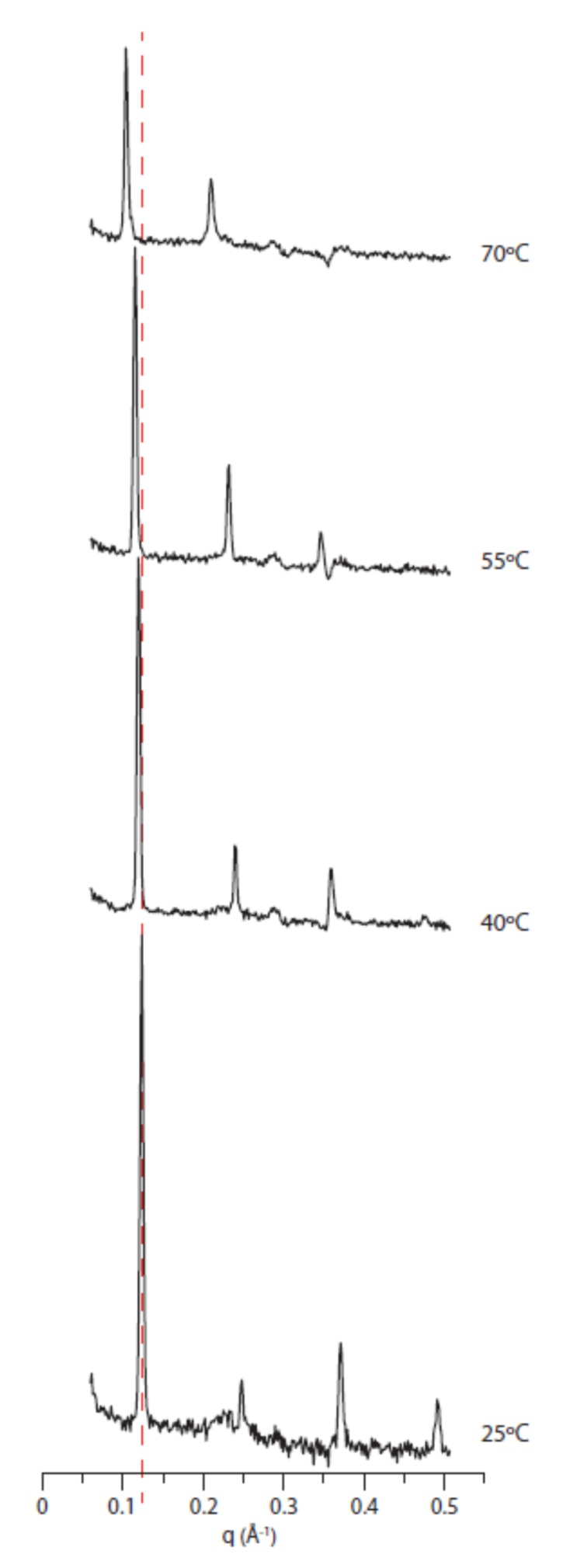
1D neutron diffractogram as a function of temperature. Neutron diffraction data DoPhPC:DoPhPE (9:1) after background subtraction obtained at 100% RH with 8% D_2_O as a function of temperature. The Y-axis intensity is (a.u.) at decimal log scale. The dotted line is guide for the eye to show the Bragg peak shifts.

The diffractogram patterns of fully hydrated and oriented DoPhPC:DoPhPE (9:1) lipid presented lamellar phases at temperatures from 25 °C up to 70 °C (Figure 4). No phase transition was observed. This is in concordance with previous research done by differential scanning calorimetry in a broad range of temperatures (−120 °C to 120 °C) [7]. Moreover, the 2D diffractograms did not show any residual non-lamellar phases (Figure S2). Previously, it was found that its ester analogue lipid DPhPC self-assembles under non-lamellar phases at temperatures above 35 °C [16]; nevertheless, it is likely that dehydration of the samples occurred when the temperature was increased. Here, since the BerILL chamber allowed an increase in temperature while carefully controlling humidity, the lamellar phase was present up to 70 °C at least.

**Figure 5:**
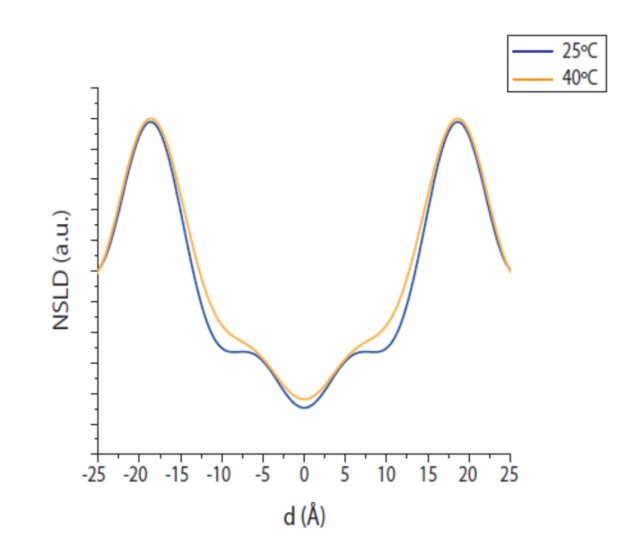
Neutron scattering length density as a function of temperature. Hydrated with 8% D_2_O. NSLD of DoPhPC:DoPhPE (9:1) obtained by neutron diffraction at 100% RH as a function of temperature: 25°C (blue) and 40 °C (ochre).

As temperature increased, the Bragg peaks’ intensities decreased due to the higher molecular motility and disorder (Figure 4). Thus, at 25 °C and 40 °C, the usual four Bragg peaks for this system were easily detectable. However, at 55 °C only three peaks were visible and at 70 °C only the first two orders from a lamellar phase were discernible. A higher number of orders resulted in a better defined NSLD, and consequently, only the NSLD obtained at 25 °C and 40 °C are comparable (Figure 5). As observed by adjusting the level of hydration, the difference between the NSLDs for both temperatures was mainly visible in the headgroup region and in a region of 10 Å on each side of the bilayer midplane, indicating a different organization of the branched chains in the lipid bilayer, also.

The lipid bilayer consisting of ether lipids and branched chains was insensitive to the change of temperature from 25 °C to 40 °C (Table 2). The bilayer thickness at 25 °C was 38.3 ± 0.2 Å and the hydrophobic region (2*D_c_*) was 32.2 ± 0.6 Å. Both values remained very similar at 40 °C. This low sensitivity to temperature can be attributed to the already existing disorder of the methyl branched chains at 25 °C. Conversely, the 2*D_c_* from lipids formed by linear acyl chains decreased at higher temperatures due to a higher thermal disorder [23].

Nevertheless, the lamellar lattice *D* increased at higher temperatures from 38.3 ± 0.2 Å at 25 °C, to 50.7 ± 0.1 Å at 25 °C and 60.0 ± 0.2 Å at 70 °C. This change of the lamellar lattice parameter may be attributed to an increase of the thickness of the water layer between lipid bilayers. For instance, *D_w_* increased by about 2 Å from 25 °C to 40 °C.

## 3. Materials and Methods 

### 3.1. Chemicals

The synthetic archaeal lipids, 1,2-di-O-phytanyl-sn-glycero-3-phosphocholine and 1,2-di-O-phytanyl-sn-glycero-3-phosphoethanolamine, were purchased from Avanti Polar Lipids Inc. (Alabaster, USA). These lipids have the same characteristics as archaeal monopolar lipids. DoPhPC is an ether lipid, to which we added a portion of DoPhPE, an electrically neutral molecule with a particular small headgroup, to mimic the mixture of lipids of the cell membrane.

### 3.2. Lipid Bilayers Formation

The lipid bilayers were studied as a multistack of hundreds of lipid bilayers on a substrate to enhance the intensity of the scattered neutrons. The substrates used were one-side polished ultraclean silicon wafers with a thickness of 275 ± 25 µm purchased from Si-Mat (Kaufering, Germany). Si wafers were rinsed with ethanol and dried by nitrogen flux. Then, 3 mg of the lipid mixture in a chloroform: methanol (2:1) solution was deposited on the Si wafer using the “rock and roll method” [26]. After the lipid mixture was deposited, the wafer with the lipid film was placed under a vacuum overnight to remove all traces of solvent. Then, the sample was annealed at 40 °C for 48 h. 

### 3.3. Neutron Diffraction

Neutron diffraction experiments were performed on multistacks of oriented membrane bilayers on the D16 small momentum transfer diffractometer [27] at the Institut Laue–Langevin (Grenoble, France) using the incident wavelength λ = 4.55 Å, and an accessible q-range from 0.06 Å^−1^ to 0.51 Å^−1^. Q is the scattering wave vector and is defined as:

$$q=\frac{2\pi sin\left(2\theta \right)}{\lambda }$$

where 2*θ* is the scattered angle. The wafer with the sample was placed on a goniometer head and rehydrated inside a humidity chamber, named BerILL, that allowed a precise control of relative humidity and temperature of the samples [28]. The wafer was placed vertically on the goniometer and correctly aligned using a laser. The Si wafer was positioned vertically parallel to the neutron beam so that the multistack of lipid bilayers on its surface was also parallel to the beam, and its angle, i.e., omega, was rotated from −0.5° to 12° by 0.05° step. The detector was placed 12° from the sample. H_2_O/D_2_O was placed in a copper bath at the chamber bottom. The BerILL included a hygrometer next to the sample to precisely measure its hydration level. For the first humidity setting, the sample was equilibrated for at least six hours and two hours of extra equilibration was used for 80%, 90%, 95%, 98%, and 100% RH. Each sample diffractogram was calibrated with a measure made with water and subtracted from the signal of the empty BerILL chamber to obtain a better signal/noise ratio. The ILL data is available at 10.5291/ILL-DATA.8-02-762 [29].

The lamellar d-spacing of the lipid bilayer was determined from the observed 2*θ* of the Bragg peak positions according to $D=\frac{2\pi }{q}$. The sum of the neutron scattering lengths per unit volume is known as a neutron scattering length density profile. The NSLD profile was calculated as a discrete set of Fourier coefficients *f_n_* according to the formula [30]:

(1) $$\rho_{bilayer}\left(z\right)=\frac{2}{D}\sum_{n=1}^{M}f_{n}\nu_{n}cos\left(\frac{2n\pi }{D}z\right)$$

where the coefficients *f_n_* for oriented samples can be found due to the formula $I_{n}=\frac{{\left|f_{n}\right|}^{2}}{q_{z}}$ as described in [31], *q_z_* is the Lorentz correction factor for oriented bilayers and *I_n_* is the integrated intensity of the *n^th^* Bragg peak, and *ν_n_* corresponds to the phase of the structure factor. As $\left|f_{n}\right|$ is a complex function, but the phase is cancelled out in the measured intensity, it was necessary to determine the phase otherwise. If the studied structure is centrosymmetric, as it was in the case of the multistack of lipid bilayers, its Fourier transform is real, and consequently, it can only be obtained by $+\left|F_{h}\right|$ or $-\left|F_{h}\right|$. To resolve the phase problem then, we used the linear correlation of the structure factors. Due to the centrosymmetry, the scattering density would be constant or would change linearly with the percentage of D_2_O in the solution. Since $z=\pm \frac{D}{2}$, the linear slope should be positive or negative. Consequently, it is possible to solve the phase by plotting the structure factors vs D_2_O content [32], which must show a linear relation. We measured the samples under a contrast of 8% D_2_O, 20% D_2_O, 50% D_2_O, and 100% D_2_O, and the order of measurement was randomized. The assigned phases for the four Bragg orders detected were −, −, +, −. For the data analysis, we used the software Origin [33].

Several parameters of the lipid bilayer can be extracted from the NSLD [20]: the Gibbs–Luzzatti bilayer thickness (*D_B_*), the hydrophobic core thickness 2*D_c_*, and the thickness of the water layers between the lipid bilayers *D_w_*. *D_B_* was represented in the NSLD profile as the distance between the two maxima and was related to the average interfacial area/lipid (A) as $D_{B}=2\frac{V_{l}}{A}$, where *V_l_* is the lipid molecular volume. The headgroup regions were fitted on the NSLD, assuming Gaussian distributions to determine their exact position in the lattice unit. Moreover, *D_c_* was related also to A as *D_c_* = *V_c_/A*, where *V_c_* is the volume of the lipid hydrophobic core. Finally, the water layer thickness was defined as $D_{w}=D-D_{B}$, and *n_w_* defined the number of water molecules. The error shown in the data was the cumulative error from the first Gaussian fit and the consequent arithmetic operations.

## 4. Conclusions

**Figure 6:**
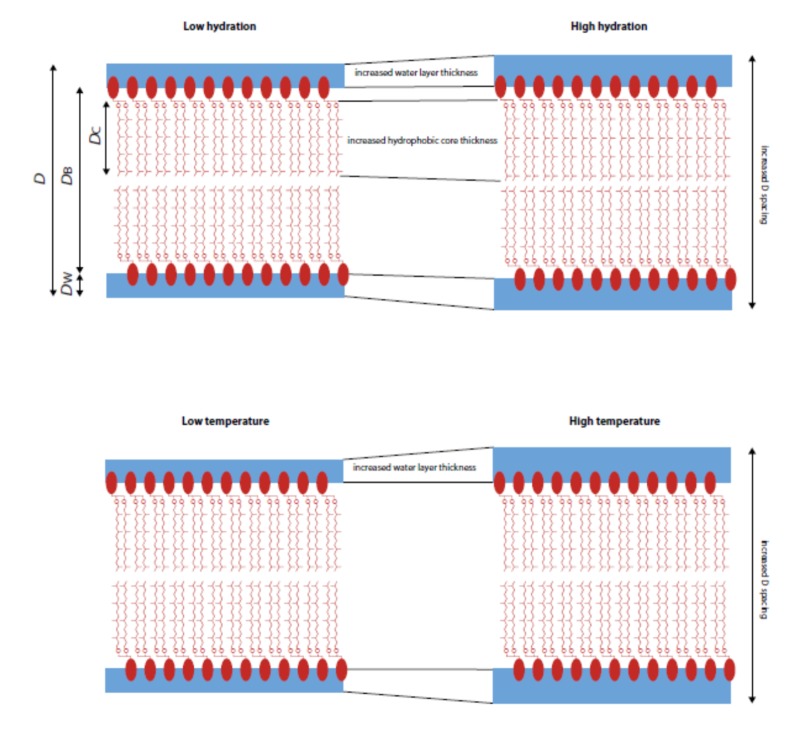
Schematic summary of the effects of hydration and temperature on the bilayer membranes.

Our aim was to study the effect of hydration and temperature on ordered multistack layers of a mixture of archaeal-like lipids. We used a mixture of DoPhPC:DoPhPE (9:1, mol:mol), which are synthetic archaeal lipids with characteristics similar to natural archaeal monopolar lipids. These synthetic lipids are available commercially (>98% purity) and served as a model, as their composition was known exactly which makes the sample preparation reproducible. We were able to determine the lipid structural data and phases at different levels of relative humidity and temperature by neutron diffraction using a precise humidity chamber. Our findings are summarized schematically in Figure 6. On one hand, hydration levels up to 80% RH displayed a system self-organized under lamellar and non-lamellar phases, while above 90% RH, only the lamellar phases were present. At 90% RH, a phase transition between two lamellar phases was clearly detected. Moreover, as observed on the NSLD, the change in hydration of the sample caused a rearrangement of its hydrophobic region consisting of the hydrocarbon chains. The increased thickness of the hydrophobic core suggests that the polyisoprenoid chains of the lipid extended more freely in the hydrophobic region, possibly because the polar region is more mobile upon higher hydration. Since both lipids have the same core regions, we could not distinguish the relative contribution of each lipid, but there was no evidence of a liquid-liquid phase separation, so it seems reasonable to assume that all lipids participated similarly whatever the temperature and hydration conditions were. On the other hand, the studied lipid system at full hydration self-assembled under lamellar phases from 25 °C to 70 °C and no phase transition was discernible. Such findings underline the rather high resistance of these specific lipids against temperature. The NSLD and structural data from the sample at 25 °C and 40 °C did not change significantly. The absence of temperature effects was explained by the already high disorder conferred by the branched chains. Thus, higher thermal disorder cannot impact more on the system. Although archaeal lipid bilayers with inositol headgroups have been shown to be less hydrated than conventional PC lipids [34], this study showed that archaeal-like lipids with PC and PE headgroups present a higher hydration than bacterial lipids. Nevertheless, we detected a modest increase of water thickness due to the temperature increase. With respect to the role of ether polyisoprenoid lipids in archaeal biology, our study is one of the first to concentrate on bilayer forming archaeal lipids. Our results clearly show that these lipids self-assemble into stable bilayers, in which thermal stability largely exceeds that of the equivalent bacterial lipids, even if we did not determine up to which extrema they would be able to resist. One could speculate that other archaeal lipids would present similar properties if they contained related headgroups, as is the case for classical lipids. It would be interesting to determine the temperature dependence of other membrane parameter values—such as permeability to protons and water, rigidity and viscosity—which have not been tested here, but are of utmost importance for cell functions. Moreover, studies with a broader distribution of lipids, or lipids extracted directly from archaeal membranes, would be mandatory to verify that our results still apply to them.

## Figures and Tables

**Table 1:** Structural parameters from DoPhPC:DoPHPE (9:1) obtained by neutron diffraction as a function of relative humidity. The samples were hydrated with 8% D_2_O. In case of 50% and 80% RH, it was not possible to extract all parameters as the membranes were not sufficiently structured to yield enough Bragg peaks.

(Å)	50% RH	80% RH	90% RH	95% RH	98% RH	100% RH
***D***	42.3 ± 0.2	45.2 ± 0.2	48.6 ± 0.1	49.5 ± 0.1	50.2 ± 0.1	50.4 ± 0.1
***D_B_***			36.5 ± 0.2	36.9 ± 0.2	37.4 ± 0.2	37.4 ± 0.2
**2*D_C_***			30.6 ± 0.6	30.8 ± 0.4	31.1 ± 0.6	31.4 ± 0.6
***D_w_***			12.1 ± 0.3	12.8 ± 0.3	13.0 ± 0.3	13.0 ± 0.3
***n_w_***			15.4 ± 0.3	16.1 ± 0.2	16.2 ± 0.2	16.2 ± 0.2

**Table 2:** Structural parameters from DoPhPC:DoPhPE (9:1) fully hydrated with 8% D_2_O obtained by neutron diffraction as a function of temperature. In case of 55 °C and 70 °C, it was not possible to extract all parameters as the membranes were not sufficiently structured to yield enough Bragg peaks.

(Å)	25 °C	40 °C	55 °C	70 °C
***D***	50.7 ± 0.1	52.5 ± 0.1	54.2 ± 0.2	60 ± 0.2
***D_B_***	38.3 ± 0.2	38.2 ± 0.2		
**2*D_C_***	32.2 ± 0.6	31.8 ± 0.6		
***D_w_***	12.6 ± 0.3	14.3 ± 0.3		
***n_w_***	15.3 ± 0.2	17.5 ± 0.2		

## Supplementary Materials

Supplementary materials can be found at https://www.mdpi.com/1422-0067/21/5/1816/s1.

## Abbreviations

DoPhPC	1,2-di-O-phytanyl-sn-glycero-3-phosphocholine
DoPhPE	1,2-di-O-phytanyl-sn-glycero-3-phosphoethanolamine
ILL	Institut Laue–Langevin
NSLD	Neutron Scattering Length Density
RH	Relative Humidity
