# Supplementary material for: Structural Characterization of an Archaeal Lipid Bilayer as a Function of Hydration and Temperature
Source: Int J Mol Sci. 2020 Mar 6;21(5):1816. doi: 10.3390/ijms21051816 (PMC7084678; doi:10.3390/ijms21051816)
Supplement: Supplementary file 1 [file ijms-21-01816-s001.pdf]

Supplementary information

Structural characterization of an archaeal-like lipid bilayer as function of hydration and temperature

Marta Salvador-Castell [1], Bruno Demé [2], Philippe Oger [1*] & Judith Peters [2,3 *]

[1] Université de Lyon, INSA de Lyon, CNRS, UMR 5240, 69211 Villeurbanne, France

[2] Institut Laue Langevin, 38000 Grenoble, France

[3] Université Grenoble Alpes, LiPhy, CNRS, 38000 Grenoble, France

* corresponding author: philippe.oger@insa-lyon.fr, jpeters@ill.fr

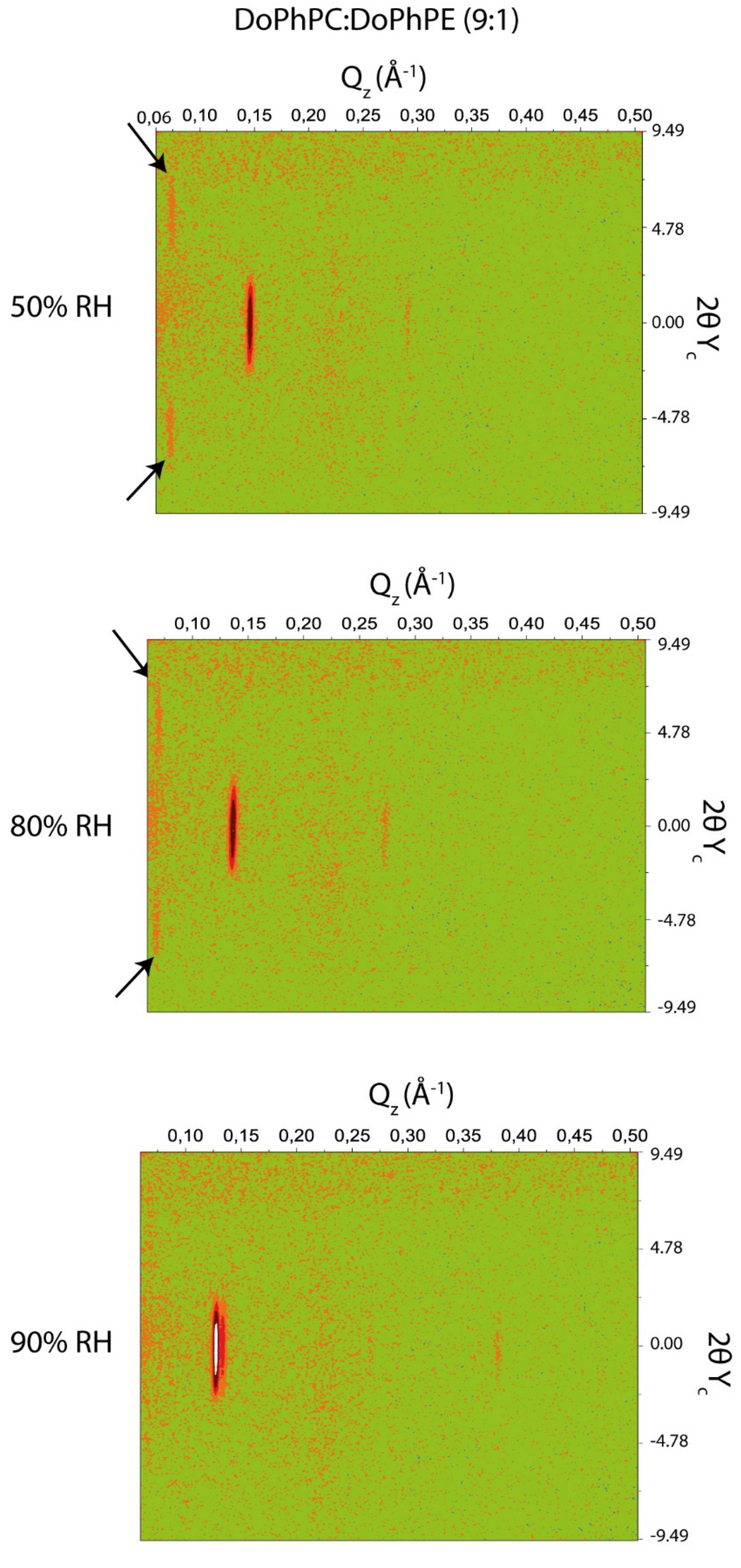

Figure S1. 2D neutron diffraction patterns of DoPhPC:DoPhPE (9:1) obtained by neutron diffraction at 25ºC as function of relative humidity. Arrows indicate the diffraction signals corresponding to non-lamellar phases.

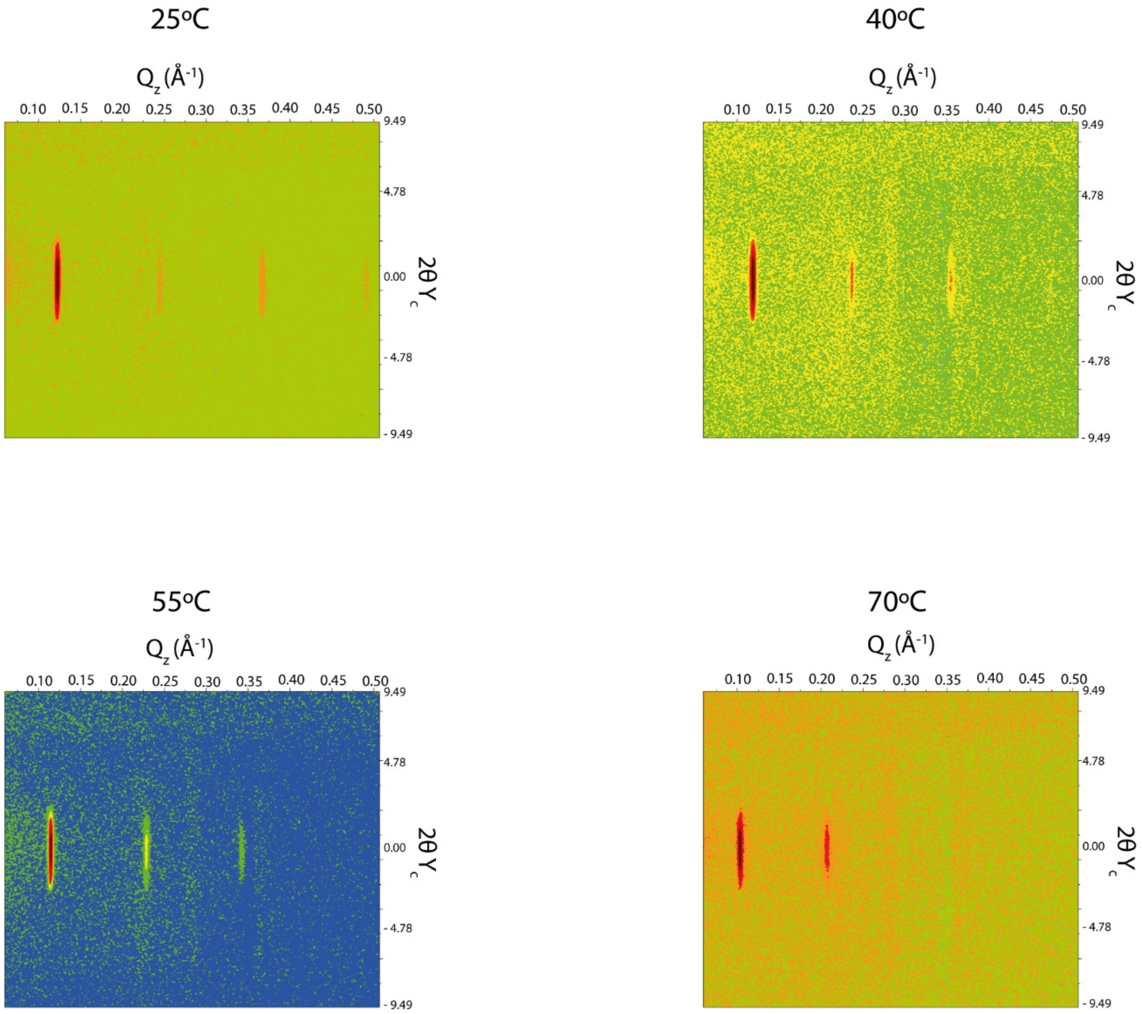

Figure S2. 2D neutron diffraction patterns of DoPhPC:DoPhPE (9:1) at 100% RH as function of temperature.
